# Strength testing of the human olfactory nerve at the frontal skull base

**DOI:** 10.1007/s10143-012-0378-1

**Published:** 2012-03-08

**Authors:** Masato Tomii

**Affiliations:** Department of Neurosurgery, Southern Tohoku General Hospital, 2-5 Satonomori Iwanuma, Miyagi, 989-2483 Japan

**Keywords:** Olfactory nerve, Anosmia, Strength, Dysfunction

## Abstract

Olfactory dysfunction may influence the quality of life tremendously. This study investigated the strength of the human olfactory nerve at the frontal skull base using cadavers. A total of 180 olfactory nerves were examined in 90 human cadaveric heads. The cut edges of the olfactory nerves were pulled until they were pulled out from the skull base. In the first set of 30 cases, each right olfactory nerve was pulled 0° laterally and 0° upward, and each left olfactory nerve was pulled 0° laterally and 15° upward. In the second set of 30 cases, each right olfactory nerve was pulled 0° laterally and 15° upward, and each left olfactory nerve was pulled 15° laterally and 15° upward. In the third set of 30 cases, each right olfactory nerve was pulled 15° laterally and 15° upward, and each left olfactory nerve was pulled 30° laterally and 15° upward. The strength of the olfactory nerve was measured when pulled in each direction. There was no significant difference in the strength of the olfactory nerves when pulling them in the postero-upward direction between 0° and 15° upward. The strengths of the olfactory nerves when pulling them in the postero-lateral direction 0° and 15° laterally were 3.14±1.87 and 4.05±1.70 g (mean ± standard deviation [SD]), respectively; the difference was almost significant. The olfactory nerve could be pulled more laterally than posteriorly because the retraction force is absorbed by the lateral wall of the olfactory fossa.

## Introduction

Olfactory dysfunction may influence quality of life tremendously. Thus, the high prevalence of olfactory dysfunction after surgery or head trauma has a great effect on overall quality of life. Following head injury with a fracture across the cribriform plate, a minor blow to the occipital area may result in anosmia due to tearing or shearing of olfactory nerve filaments. The olfactory nerve and tracts are also at risk during intradural explorations using the interhemispheric approach or the frontotemporal approach [10, 11]. Both approaches require some degree of frontal lobe retraction, which may result in temporary or permanent olfactory dysfunction because of nerve avulsion or mechanical compression [3, 4, 6, 12]. A bilateral olfactory nerve deficit is unusual after a unilateral approach [9]. Furthermore, it is well known that bilateral anosmia is highly likely to occur when bilateral olfactory nerves are pulled out from the frontal base. It is not always true that preservation of the anatomical structure will result in preservation of function. However, preservation of anatomical structure may be an important factor in the preservation of olfactory function. Based on this background, strength tests of the human olfactory nerve at the frontal skull base were performed using cadavers, and the microsurgical anatomical features of this nerve were reviewed in order to identify operative nuances that may contribute to reducing the rate of postoperative olfactory dysfunction.

## Methods

**Fig. 1:**
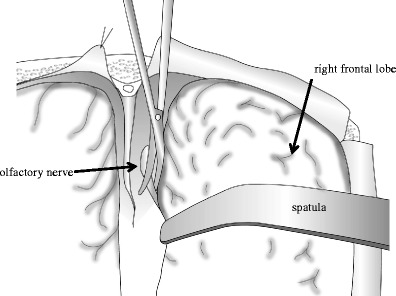
Schematic drawing view of the technique of olfactory nerve dissection

**Fig. 2:**
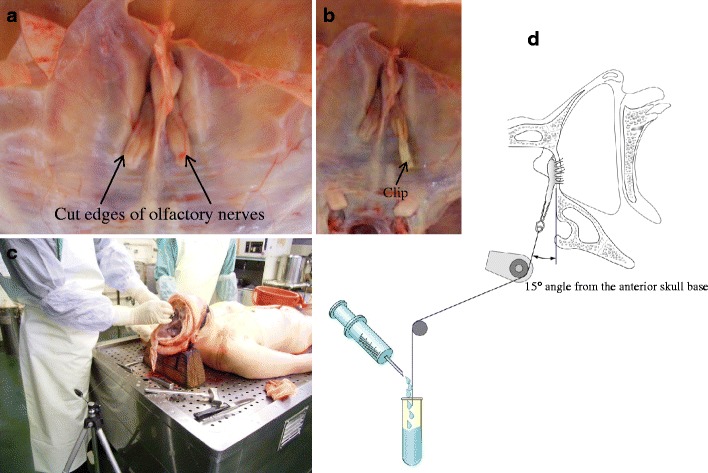
**a** Superior view of the anterior skull base. Bilateral olfactory nerves have been severed 10–15 mm from the skull base. **b** The cut edges of the olfactory nerve are clipped and connected to a light, small rubber bag by a monofilament string. Whole view (**c**) and schematic drawing view (**d**) of this study. The angle of pulling of the olfactory nerve from the frontal skull base and from the axis of the olfactory nerve is regulated by pulling. The olfactory nerve was pulled by a rubber bag through a string during which water was poured into the bag at 1 ml per 10 s until the nerve was pulled out from the cribriform plate

**Fig. 3:**
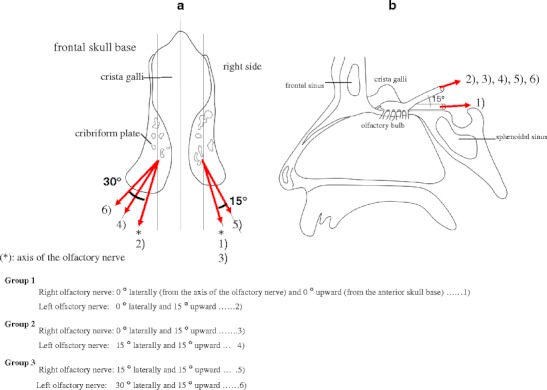
The supine (**a**) and sagittal (**b**) schematic drawing views of the anterior skull base. Numbers show the direction of pulling of the olfactory nerves in each group

All procedures were conducted in accordance with the institutional guideline of Jikei University and the Tokyo Medical Examiner’s Office. I used fresh cadavers. Ninety brains were obtained from autopsies of 68 men and 22 women, aged 24–80 years at the time of death. They were within 12 h after death and had no intracranial disease or trauma. The calvaria was opened, and the brain was removed en bloc after the olfactory nerves were severed 10–15 mm from the skull base with minimal retraction (Figs. 1 and 2a). The calvaria was positioned so that the frontal base was perpendicular to the ground without rotation. Then, the cut edges of the olfactory nerves were clipped and connected to a light, small rubber bag by a monofilament string (Fig. 2b). In the first set of 30 cases (Group 1), each right olfactory nerve was pulled 0° laterally (from the axis of the olfactory nerve) and 0° upward (from the anterior skull base) (1)), and each left olfactory nerve was pulled 0° laterally and 15° upward (2)). In the second set of 30 cases (Group 2), each right olfactory nerve was pulled 0° laterally and 15° upward (3)), and each left olfactory nerve was pulled 15° laterally and 15° upward (4)). In the third set of 30 cases (Group 3), each right olfactory nerve was pulled 15° laterally and 15° upward (5)), and each left olfactory nerve was pulled 30° laterally and 15° upward (6)). The pulling directions of the olfactory nerves are outlined in Fig. 3. The angle of pulling against the frontal skull base and the axis of the olfactory nerve was regulated by pulling. The olfactory nerves were pulled by a rubber bag through a string while water was poured into the bag at a rate of 1 ml per 10 s until the nerve was pulled out from the cribriform plate. Then, the string, clip, and the rubber bag filled with water were weighed, and this value was used as the strength of the olfactory nerve (Fig. 2c, d).

Statistical analysis was performed using the Mann–Whitney *U*-test for comparisons of the nonparametric variables with a probability value of 0.05 indicating significance.

## Results

**Fig. 4:**
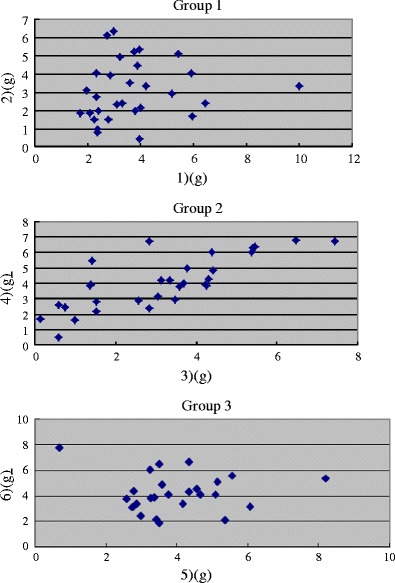
Graphs showing the strength of pulling for different directions in each group

In Group 1, the strengths (mean ± standard deviation [SD]) of the olfactory nerve for (1) and (2) were 3.68±1.74 and 3.09±1.60 g, respectively. The difference between the strengths of (1) and (2) was not significant (*p* = 0.191, Mann–Whitney *U*-test for comparisons of the nonparametric variables). In Group 2, the strengths for (3) and (4) were 3.14±1.87 and 4.05±1.70 g, respectively. The difference between the strengths of (3) and (4) was almost significant (*p* = 0.068, Mann–Whitney *U*-test for comparisons of the nonparametric variables). In Group 3, the strengths for (5) and (6) were 4.00±1.44 and 4.20±1.65 g, respectively. The difference between the strengths for (5) and (6) was not significant (*p* = 0.684, Mann–Whitney *U*-test for comparisons of the nonparametric variables) (Fig. 4; Table 1).

**Table 1:** Olfactory nerve strength

	(1)^a^ g	(2) g	(3) g	(4) g	(5) g	(6) g
Mean	3.68	3.09	3.14	4.05	4.00	4.20
SD	1.74	1.60	1.87	1.70	1.44	1.65
Min.	1.70	0.46	0.12	0.51	0.69	1.41
Max.	10.00	6.35	7.46	6.79	8.20	7.72

*SD* standard deviation, *Min.* minimum, *Max.* maximum

^a^Numbers in parentheses indicate the strength of the olfactory nerve measured as defined in Fig. 3: (1) — pulled 0**°** laterally (from the axis of the olfactory nerve) and 0**°** upward (from anterior skull base); (2), (3) — pulled 0**°** laterally and 15**°** upward; (4), (5) — pulled 15**°** laterally and 15**°** upward; (6) — pulled 30**°** laterally and 15**°** upward

**Fig. 5:**
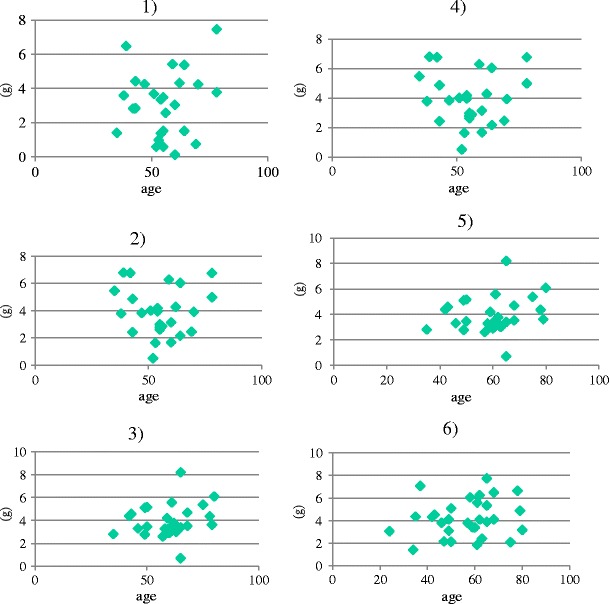
Age (*x*-axis) and strength of pulling for different directions from (1) to (6) (the *y*-axis)

And in each subgroups of pulling for different directions from (1) to (6), there seemed to be no relationship between age and olfactory strength (Fig. 5). Moreover, there were no statistical differences of olfactory strength between male and female in each subgroup.

## Discussion

The results of this study indicated that frontal lobe retraction in a posterior direction from the olfactory bulb, regardless of angle from the skull base posteriorly, is associated with the risk of pulling out the olfactory nerve, while retraction in a lateral direction appears to be relatively safe. This was based on the fact that that there was no significant difference in the strength of the olfactory nerves pulling them in a postero-upward direction between 0° and 15° upward (each, 0° laterally). Furthermore, the difference in the strengths of the olfactory nerves when they were pulled in a postero-lateral direction between 0° and 15° laterally (each, 15° upward) was almost significant.

The exact site of injury in the olfactory pathways that produces anosmia is controversial. It had been thought that only serious injuries of the head with fractures of the anterior cranial fossa resulting in anatomical disruption of the olfactory filaments or tract cause permanent anosmia, but permanent anosmia can result from even trivial injuries [11]. Previous studies have reported anosmia after surgical treatment of anterior communicating artery (ACoA) aneurysms and suggest that its prevalence depends on the mode of approach to the aneurysm [6]. It has been mentioned that there are three mechanisms of olfactory nerve damage during frontal lobe retraction (depression and elevation): partial or total avulsion of the olfactory nerve from the cribriform plate [1–4, 6, 9]; injury during nerve dissection; and direct pressure damage [2, 9]. Cardali et al. [2] suggested that maintaining the anatomic integrity of the nerve is important to preserve olfactory function. Even a mild retractive pressure on the nerve can lead to temporary or permanent lesions [2, 6]. It is said that this is because the microvasculature lying on the dorsal surface of the nerve, which is constituted of microscopic branches of the ethmoidal arteries nourishing the nerve, is fragile to retraction and compression of the frontal lobe. Suzuki at al. [12] reported that 42% of patients with bilateral preservation of the olfactory nerves in bifrontal craniotomy for anterior communicating artery aneurysms developed anosmia. They also reported that one-third of the patients who had both olfactory tracts damaged or severed during surgery had normal olfactory function. Thus, they mention that many uncertainties concerning olfaction in general remain. Invisible factors affecting the olfactory nerve, such as heat injury to the nerve by drilling the bone surrounding the nerve or direct pressure damage to the medial frontal lobe (septal area) by retraction, might cause anosmia [7]. Furthermore, Vries at al. [13] reported no improvement of olfactory nerve function at follow-up after having olfactory nerve damage after the operation, which indicates that the regenerative capacity of the olfactory nerve is limited. Thus, in cases in which a mechanical disruption (neurotmesis) of the fila olfactoria has occurred, regeneration would not be expected. On the other hand, Rouit and Murali [11] noted that traumatic anosmia may recover at any time from a few days up to 5 years.

**Fig. 6:**
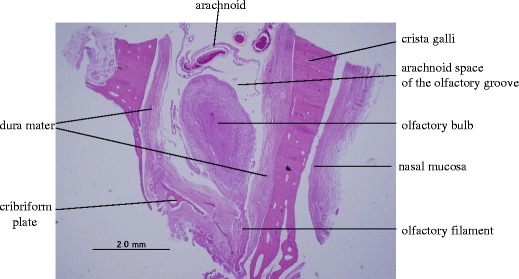
Histological study of the olfactory fossa in the coronal view demonstrating that there is a large lateral wall bilaterally at any part of the olfactory fossa. Hematoxylin and eosin (H&E) stain, original magnification ×20

However, in our research, we have focused only on maintaining the anatomic integrity of the olfactory nerve. Anatomically, the olfactory bulb is located on the cribriform plate of the olfactory fossa, which is a couple of millimeters lower than the surrounding cranial base. The depth of the olfactory fossa is 4.8 (0.6–11.7) mm in the anterior part, 5.0 (0–15.5) mm in the middle part, and 3.2 (0–10.0) mm in the posterior part [5]. Thus, the olfactory filaments are fairly well protected in the cribriform plate against the shearing stresses of the brain that often accompany even minor injuries to the head [5]. From our research, the olfactory nerve could easily be pulled out irrespective of the degrees of pulling from the frontal base towards the posterior direction. However, the olfactory nerve could withstand more force when pulled laterally against the axis of the olfactory nerve. This is because there is only a tiny bone in the posterior wall (planum sphenoidale, frontal edge) compared to the anterior wall of the olfactory fossa. On the other hand, there is a large lateral wall bilaterally along the entire olfactory fossa (Fig. 6). Thus, it might hold the nerve in the cribriform plate when it is pulled laterally. There is a possibility that the pulling force is absorbed by the lateral wall of the olfactory fossa when the nerve is pulled laterally. However, there was no significant difference in the strength of the olfactory nerve between pulling the nerve 15° and 30° laterally. Therefore, with frontal lobe retraction in a posterior direction from the olfactory bulb, the olfactory nerve is at high risk of being pulled out, but in a lateral direction, retraction is relatively safe from the perspective of maintaining the anatomical integrity of the olfactory nerve. However, if the nerve is pulled more laterally, it is pressed harder against the bone of the lateral wall of the olfactory fossa and is twisted against the axis of this nerve. These factors could, in turn, damage this nerve. But these data should be considered in realistic surgical settings like bifrontal approach and pterional approach, and one should try to measure the strength of olfactory nerve drawn by spatula pressure and of the own weight of the brain shifting to get a better clinical impact.

Several techniques have been reported to prevent perioperative olfactory nerve injury [1, 2, 4, 9, 12]. Most of them are techniques that minimize the effect on the olfactory nerve from frontal lobe retraction during surgery. Nakayama [8] recommended that frontal lobe retraction should be done towards the superficial direction to put less tension on the olfactory nerve. Fujiwara et al. [4] and Suzuki at al. [12] reported that, to prevent nerve injury on the operated side, dissection of the nerve at the beginning of the microsurgical procedures is recommended, and the key to successful separation of the olfactory tract from the brain is never to apply pressure in a downward direction posteriorly from the olfactory bulb, but to continually apply upward pressure (toward the olfactory bulb) on the frontal lobe to which the tract is adherent [12]. These techniques are supported by our results. Aydin et al. [1] reported that olfactory nerve function could be preserved at a relatively high rate of 85%. This high rate probably resulted from the microtechnique used during the relatively cautious frontal lobe retraction, which was less than 1.5 cm.

In addition, the nerve is pulled out by a strength of only 3–4 g. The present study had some limitations because, during the actual operation, the nerves are pulled while retracting the brain. Thus, these data of strength alone do not provide definitive information that would be useful during operation. If preoperative coronal CT scanning is done, and the surrounding bone structure of the cribriform plate is investigated, that is, the depth of the olfactory fossa from the surrounding anterior skull base in every direction, which direction the olfactory nerve can be pulled to minimize damage to the nerve as much as possible can be identified.

In each group, the absolute value of the strength of the olfactory nerves varied widely. This difference may come from not only the direction of pulling the nerve, but also from gender, aging, the length of time after death, and other factors.

## Conclusion

This study found that frontal lobe retraction in a posterior direction may be dangerous from the point of view of the postoperative prevalence of anosmia, but retraction in a lateral direction may be relatively safe.
